# Human Domain Antibodies to Conserved Epitopes on HER2 Potently Inhibit Growth of HER2-Overexpressing Human Breast Cancer Cells In Vitro

**DOI:** 10.3390/antib8010025

**Published:** 2019-03-18

**Authors:** Hongqian Wang, Yanping Wang, Zuoxiang Xiao, Wei Li, Dimiter S. Dimitrov, Weizao Chen

**Affiliations:** 1 The Second Clinical Medicine School, Zhejiang Chinese Medical University, Hangzhou 310005, China; 15990116059@163.com; 2 Zhejiang Shimai Pharmaceutical Co., Ltd., Hangzhou 311100, China; zxiao@centrymed.com; 3 CentryMed Pharmaceutical Inc., a subsidiary of Zhejiang Shimai Pharmaceutical Co., Ltd., Frederick, MD 21704, USA; wangyp18@centrymed.com; 4 Center for Antibody Therapeutics, Department of Medicine, School of Medicine, University of Pittsburgh, Pittsburgh, PA 15261, USA; liwei171@pitt.edu (W.L.); dsd116@pitt.edu (D.S.D.)

**Keywords:** HER2, cancer, therapy, domain antibody, conserved epitope

## Abstract

The FDA approval of two anti-HER2 monoclonal antibodies, trastuzumab and pertuzumab, and an antibody-drug conjugate, trastuzumab emtansine, has transformed clinical practice for HER2-positive cancers. However, not all patients respond to therapy, and the majority of responders eventually develop resistance. In addition, cardiotoxicity is a major safety concern for their clinical use. Thus, there remains a need for the discovery and development of novel classes of HER2-targeted therapeutics with high efficacy and specificity. In this study, we report the identification and characterization of fully human single-domain antibodies (dAbs) targeting HER2 epitopes that are highly conserved among various species and non-overlapping with those of trastuzumab and pertuzumab. An Fc-fusion protein of the best binder demonstrated much higher inhibitory activity against HER2-amplified human breast cancer cell lines than trastuzumab and pertuzumab. Unlike the latter, however, it did not have an effect on gastric and ovarian cancer cell lines with HER2 overexpression. The dAb-Fc fusion protein showed poor pharmacokinetics in mice, thus limiting its potential for therapeutic use. It could be useful as an agent for the exploration of functionally important conserved structures on HER2 with implications for the design of novel therapeutics and elucidation of mechanisms of HER2-mediated tumorigenesis.

## 1. Introduction

Human epidermal growth factor receptors (HER, also known as ErbB) are tyrosine kinases composed of four extracellular domains, a single transmembrane segment, and an intracellular cytoplasmic domain with a number of tyrosine phosphorylation sites [1]. They are involved in the regulation of various cellular functions and their aberrant expression and activation have been implicated in tumorigenesis and the progression of a number of cancers. In mammalian cells, the ErbB system contains four members, including ErbB1 (EGFR), ErbB2 (HER2), ErbB3 (HER3), and ErbB4 (HER4), and at least twelve ligands, such as epidermal growth factor (EGF) and heregulin [2,3]. Ligand binding induces conformational changes of ErbB from a “closed” state to an “open” state leading to dimerization of the receptors and downstream signaling. HER2 is the only ErbB member for which no ligand has been found. However, unliganded monomeric HER2 naturally adopts an “open” conformation and can form homodimers and heterodimers with three other family members [4]. 

HER2 overexpression and gene amplification have been observed in a fraction of breast cancer [5], gastric cancer [6], ovarian cancer [7], non-small cell lung cancer [8], and other cancers [9]. Trastuzumab (Herceptin), an FDA-approved humanized monoclonal antibody (mAb) targeting the extracellular domain IV of HER2, inhibits cancer cell proliferation by mediating antibody-dependent cellular cytotoxicity (ADCC), preventing HER2 cleavage into a constitutively active form, and triggering HER2 internalization and degradation [10]. Pertuzumab (Perjeta), another FDA-approved humanized mAb to the extracellular domain II of HER2, blocks HER2 dimerization with other ErbB family members, thus inhibiting tumor growth and progression [11]. Although these HER2-targeting therapies have brought significant clinical benefits, not all patients respond. Moreover, the vast majority of responders eventually relapse. In addition to the high level of intratumoral heterogeneity of HER2 expression, truncation of HER2 extracellular domains, alteration of related intracellular proteins, and overexpression of other tyrosine kinase receptors are among the mechanisms that account for resistance to therapy [10]. Due to synergistic effects resulting from complementary modes of action, trastuzumab and pertuzumab in combination exert more effective antitumor activity than either alone [12]. A mixture of three antibodies targeting three different extracellular domains of HER2 are superior to trastuzumab, pertuzumab, and their combination, and are capable of overcoming resistance to trastuzumab [13].

Engineered single-domain antibodies (dAbs) have emerged as a novel class of candidate therapeutics against HER2-expressing cancers [14]. Such dAbs are of relatively small molecular size (11–15 kDa) and, therefore, can target cryptic epitopes and antigens in obstructed locations that are not or less accessible to large-size antibodies [15,16]. A recent study demonstrated that an Fc-fusion protein of a camelid-derived dAb, C3-Fc, was better able to induce ADCC against HER2-expressing breast, colon, and ovarian cancer cell lines than trastuzumab [17]. In this study, we describe the identification and characterization of fully human dAbs targeting HER2 epitopes that are highly conserved among various species and do not overlap with those of trastuzumab and pertuzumab. The best binder was more potent than trastuzumab and pertuzumab in inhibiting HER2-overexpressing human breast cancer cells tested. These results suggest the existence of novel epitopes on HER2 that are functionally more important for breast cancer tumorigenesis than those already identified and can be targeted by antibody-based therapy. 

## 2. Materials and Methods

### 2.1. Cells, Proteins, Plasmids, and Other Reagents

We purchased 293 FreeStyle (293FS) cells and protein A agarose from Thermo Fisher Scientific. Recombinant human (hHER2), cynomolgus (cHER2), mouse (mHER2), and rat HER2 (rHER2) and other ErbB family member proteins were purchased from Sino Biological (Beijing, China). The pWC1 vector for phage display and bacterial expression and the pDin1 vector used for mammalian expression were designed and generated in our laboratory. Horseradish peroxidase (HRP)-conjugated goat anti-human IgG (Fc-specific) antibody and HRP-conjugated mouse anti-FLAG tag antibody were products of Sigma (St. Louis, MO, USA). Anti-His-phycoerythrin (PE) conjugate and goat anti-human IgG (Fc-specific)-FITC conjugate were purchased from Miltenyi Biotec (Bergisch Gladbach, Germany) and Sigma (St. Louis, MO, USA), respectively.

### 2.2. Panning and Screening of a Phage-Display Human Antibody Heavy Chain Variable Domain (VH)-Based dAb Library for Identification of HER2 Antibodies

A large phage-display human VH library was used to select dAbs against hHER2 conjugated to magnetic beads (Dynabeads M-270 epoxy; DYNAL Inc., New York, NY, USA), as described previously [18] except that we used 5, 1, and 0.1 µg of antigen in the first, second, and third rounds of panning, respectively. Individual clones bound to the antigen were identified from the third round of biopanning by using soluble expression-based monoclonal ELISA (semELISA) as described previously [19].

### 2.3. Cloning of Fc-Fusion Proteins of Anti-HER2 dAbs

We used the following primers: 

Her2F1, 5′-GATCGGCCCAGCCGGCCGAGATGCAGCTGGTGGAG-3′ (sense); 

Her2R1, 5′-GTCACAAGATTTGGGCTCTGAGGAGACGGTGACCAG-3′ (antisense);

Her2F2, 5′-GATCGGCCCAGCCGGCCGAGGTGCAGCTGGTGGAG-3′ (sense);

FcF1, 5′-GAGCCCAAATCTTGTGACAAAACTCACACATGC-3′ (sense); 

FcR1, 5′-GATCGTTTAAACTCATTTACCCGGAGACAGGGA-3′ (antisense).

For cloning of SMET2.1Fc, the SMET2.1 and human IgG1 Fc gene fragments were PCR-amplified with primer pairs Her2F1/Her2R1 and FcF1/FcR1, respectively. The two gene fragments were linked to each other by overlapping PCR with the two gene fragments in the same molarities for seven cycles in the absence of primers and 15 additional cycles in the presence of primers Her2F1 and FcR1. The full-length SMET2.1-Fc gene fragment was digested with SfiI and PmeI restriction enzymes and cloned into the pDin1 vector. SMET2.5Fc and SMET2.14Fc were generated the same way except for the use of Her2F2 and Her2R1 as primers for the amplification of SMET2.5 and SMET2.14 gene fragments, and the use of Her2F2 and FcR1 as primers for overlapping PCR.

### 2.4. Protein Expression and Purification

dAbs were expressed in *E. coli* HB2151 cells, and Fc-fusion proteins were expressed in 293FS cells as described previously [15]. His-tagged dAbs were purified from the soluble fraction of HB2151 periplasm by using the Ni-NTA resin (Qiagen, Hilden, Germany) according to the manufacturer’s protocol. Fc-fusion proteins were purified from the 293FS culture supernatants by using Protein A Sepharose 4 Fast Flow column chromatography (GE Healthcare, Chicago, IL, USA) according to the manufacturer’s instructions. 

### 2.5. Size-Exclusion Chromatography

A Superdex200 10/300 GL column (GE Healthcare, Chicago, IL, USA) was calibrated with protein molecular mass standards of carbonic anhydrase (29 kDa), ovalbumin (44 kDa), conalbumin (75 kDa), aldolase (158 kDa), and ferritin (440 kDa). Purified proteins at a concentration of 1 mg mL^−1^ in PBS (pH 7.4) were loaded onto the pre-equilibrated column and eluted with PBS (pH 7.4) at 0.5 mL/min. 

### 2.6. ELISA

ELISA was performed according to standard protocols. Briefly, recombinant antigens were coated on Corning EIA/RIA high-binding 96-well plates at 50 ng per well overnight at 4 °C and blocked with 3% nonfat milk in PBS (pH 7.4). The fivefold serially diluted antibody was added and incubated at room temperature for 2 h. The plates were washed with PBS containing 0.05% Tween 20. Bound dAbs were detected by HRP-conjugated anti-FLAG tag antibody. Bound Fc-fusion proteins were detected by HRP-conjugated goat anti-human IgG (Fc-specific) antibody. The assay was developed at room temperature with TMB substrate (Sigma-Aldrich, St. Louis, MO, USA) and monitored at 450 nm with a microplate reader. The half-maximal binding (EC_50_) was calculated by fitting the data to the Langmuir adsorption isotherm.

To measure the competitive binding of dAbs, SMET2.1 or SMET2.5 concentrations were kept constant (1.5 µg mL^−1^). The concentrations of competitors started from 100 µg mL^−1^ and were fivefold serially diluted. Bound SMET2.1 or SMET2.5 were detected by HRP-conjugated anti-FLAG tag antibody.

To measure the competitive binding of SMET2.1 with trastuzumab and pertuzumab IgG1, the concentration of SMET2.1 was kept constant (2 µg mL^−1^). The concentrations of the competitors started from 1 µM and were fivefold serially diluted. Bound SMET2.1 was detected by HRP-conjugated anti-FLAG tag antibody.

### 2.7. Flow Cytometry

About 5 × 10^5^ cells were incubated with 1.5 (for dAb) or 0.8 (for dAb-Fc or IgG1) µg mL^−1^ antibody on ice for 30 min. The cells were washed twice with PBS containing 0.1% bovine serum albumin (PBSA) and resuspended in 200 µL PBSA. Then, 2 µL anti-His-PE conjugate (for dAb) or goat anti-human IgG (Fc-specific)-FITC conjugate (for dAb-Fc or IgG1) was added and incubated for 30 min. The cells were washed twice with PBSA and then used for flow cytometry analysis.

### 2.8. In Vitro Cell Growth Inhibition Assay

First, 10^3^ cells were inoculated in each well of 96-well plates in the absence or presence of anti-HER2 antibodies of which the concentrations started from 100 nM and were fivefold serially diluted. Then, after 5–7 days, cell proliferation was measured with Promega (Madison, WI, USA) CellTiter 96^®^ AQueous One Solution Cell Proliferation Assay System according to the manufacturer’s instructions. Cell viability was calculated by using the following formula: (average reading of antibody treated group − average reading of medium only group)/(average reading of PBS treated group − average reading of medium only group).

### 2.9. Superimposition of Crystal Structures

The crystal structures of HER2-trastuzumab (Protein Data Bank entry 1N8Z) and HER2-pertuzumab complexes (Protein Data Bank entry 1S78) together with the PyMOL molecular graphics system (version 1.5.0.4; Schrödinger, LLC) were used for superimposition and representation of variable regions of HER2 among different species. 

### 2.10. Pharmacokinetic Measurement in Mice

NOD/SCID mice were administered intravenously with 0.5 mg antibody on day 0. Plasma samples were collected on days 1, 3, 5, and 6. Antibody concentrations were determined by ELISA with standard curves generated using the original antibody stocks. The study was performed under protocols approved by the Zhejiang Chinese Medical University Animal Care and Use Committee.

## 3. Results

### 3.1. Selection of Anti-HER2 dAbs

**Figure 1:**
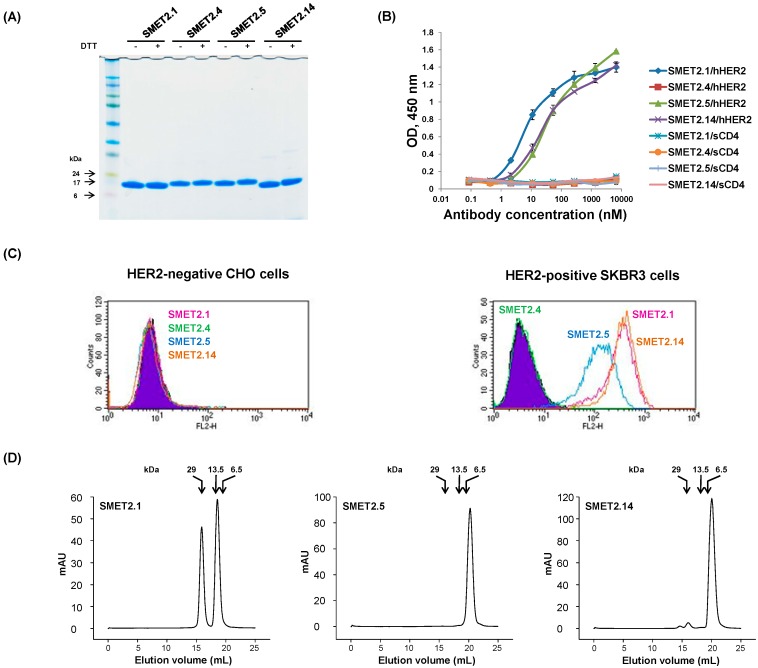
Identification and initial characterization of anti-HER2 dAbs. (**A**) Non-reducing and reducing SDS-PAGE of selected anti-HER2 dAbs. Molecular masses of standards are shown on the left. (**B**) ELISA binding of the anti-HER2 dAbs to hHER2 and sCD4, an irrelevant antigen. Antigens were coated on 96-well plates at a concentration of 2 µg mL^−1^. Bound dAbs were detected by horseradish peroxidase (HRP)-conjugated anti-FLAG tag antibody. The assay was performed in duplicate. Results are the mean ± standard deviation. (**C**) Flow cytometry analysis of the dAbs. Diagrams for reference cells are filled with purple. Diagrams for cells incubated with SMET2.1, SMET2.4, SMET2.5, and SMET2.14 are in pink, green, blue, and yellow, respectively. (**D**) Size-exclusion chromatography of the dAbs. The arrows at the top indicate the elution volumes of molecular mass standards in PBS (pH 7.4).

We have recently constructed a large (size, 3 × 10^11^) phage-display human VH dAb library with peripheral blood B cells from approximately 200 healthy individuals according to previously published protocols [20,21]. Highly diversified, naturally occurring complementarity-determining regions 2 (CDR2) and 3 (CDR3) of human antibody heavy chains were grafted into a human VH3-23 germline-based framework (FR) scaffold and four putative solvent-accessible residues (residues 27, 29, 31 and 32; IMGT numbering scheme) in the CDR1 of the scaffold were randomly mutated. The library was used for panning and screening for high-affinity binders with hHER2 protein. Four dAbs, designated SMET2.1, SMET2.4, SMET2.5, and SMET2.14, were identified, which were expressed at high levels (52, 58, 50, and 71 mg L^−1^, respectively) in bacteria (Figure 1A). All except SMET2.4 bound to hHER2 with SMET2.1 having the highest affinity in an ELISA (Figure 1B). None of the dAbs interacted with a soluble form of human T cell receptor CD4 (sCD4). At a concentration of 100 nM, they strongly bound to the HER2-overexpressing human breast cancer cell line SKBR3 but not to Chinese hamster ovary (CHO) cells, suggesting high specificity of the antibodies (Figure 1C). Size-exclusion chromatography analysis revealed that about 40% of SMET2.1 existed as a dimer with an apparent molecular weight (aMW) of approximately 30 kDa (Figure 1D). SMET2.5 was a monomer while a small amount of dimer was also observed with SMET2.14. The monomers of SMET2.5 and SMET2.14 were eluted more slowly than expected with an aMW (<6.5 kDa) smaller than their calculated molecular weights (cMW) (15.7 and 14.7 kDa, respectively, including the hexahistidine and FLAG tags at the C terminus) likely due to weak transient interactions with the column matrix, as observed in a previous study [19]. 

### 3.2. Generation and Initial Characterization of Fc-Fusion Proteins of the Anti-HER2 dAbs

**Figure 2:**
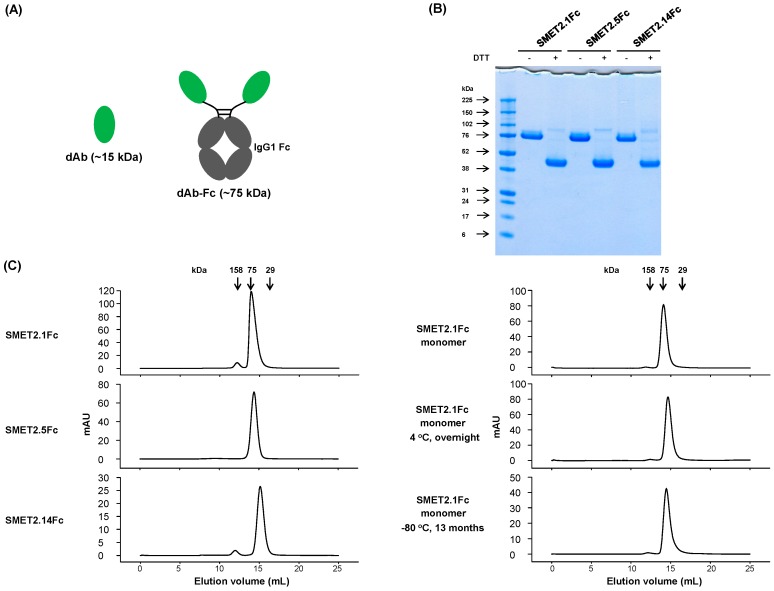
Generation and initial characterization of Fc-fusion proteins of anti-HER2 dAbs. (**A**) Schematic representation of dAb-Fc fusion proteins. Estimated molecular weights of dAbs and dAb-Fc fusion proteins are shown in parenthesis. (**B**) Non-reducing and reducing SDS-PAGE of the dAb-Fc fusion proteins. Molecular masses of the standards are shown on the left. (**C**) Size-exclusion chromatography of the dAb-Fc fusion proteins. The arrows at the top indicate the elution volumes of the molecular mass standards in PBS (pH 7.4).

**Figure 3:**
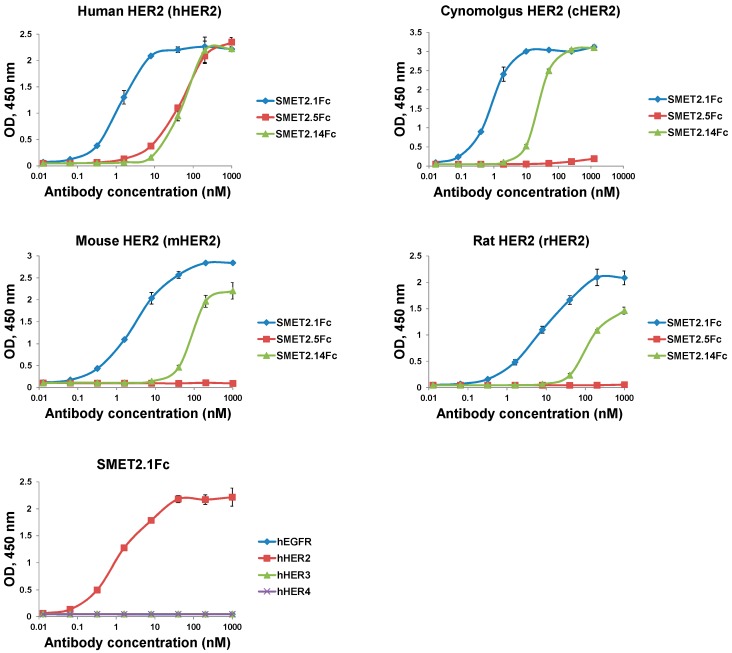
ELISA binding of the dAb-Fc fusion proteins to recombinant HER2 proteins from different species or different human ErbB family member proteins. Recombinant antigens were coated on 96-well plates at a concentration of 2 µg mL^−1^. Bound Fc-fusion proteins were detected by HRP-conjugated goat anti-human IgG (Fc-specific) antibody. The assay was performed in duplicate. Results are the mean ± standard deviation.

To evaluate the potential of the dAbs to be developed as candidate therapeutics, we fused them to human IgG1 Fc, which could improve stability and confer bivalency (avidity) and a long half-life in vivo (Figure 2A). The Fc-fusion proteins, designated SMET2.1Fc, SMET2.5Fc, and SMET2.14Fc, were expressed and purified from transiently transfected 293FS cell culture supernatants by using protein A with a yield of 10–20 mg L^−1^ (Figure 2B). Size-exclusion chromatography revealed that the vast majority (>90%) of the purified fusion proteins in PBS (pH 7.4) were monomers with an aMW of approximately 80 kDa, comparable to their cMWs (Figure 2C, left panel). Elution of SMET2.14Fc monomer was delayed, as observed with the dAb (Figure 1D), likely due to weak transient interactions with the column matrix. In an ELISA, SMET2.1Fc and SMET2.14Fc were cross-reactive against hHER2, cHER2, mHER2, and rHER2, whereas SMET2.5Fc bound only to hHER2 (Figure 3). SMET2.1Fc had the highest binding activity with EC_50_s of approximately 1.3, 0.81, 3.1, and 8.2 nM for hHER2, cHER2, mHER2, and rHER2, respectively. In a parallel experiment, we found that SMET2.1Fc did not interact with recombinant human EGFR (hEGFR), HER3 (hHER3), or HER4 (hHER4) proteins, suggesting high specificity of the antibody for HER2 (Figure 3). 

### 3.3. Growth Inhibition of HER2-Overexpressing Human Cancer Cell Lines by the dAb-Fc Fusion Proteins

**Figure 4:**
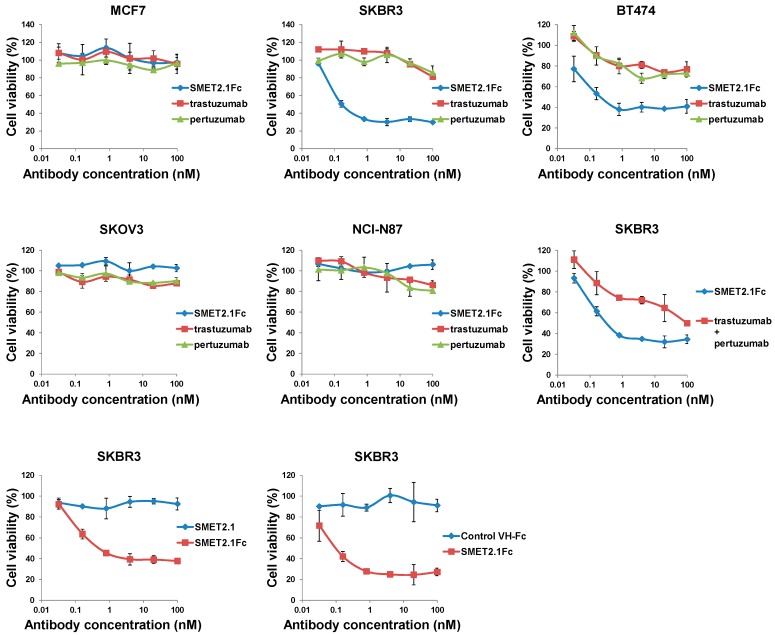
Growth inhibition of HER2-expressing human cancer cell lines in cell cultures. Trastuzumab and pertuzumab used in this experiment are full-length IgG1 antibodies. The assay was performed in duplicate. Results are the mean ± standard deviation.

In a preliminary experiment, we found that all three Fc-fusion proteins were able to potently inhibit the proliferation of the HER2-overexpressing human breast cancer cell line SKBR3 in a dose-dependent manner with SMET2.1Fc having the highest inhibitory activity (Figure S1). SMET2.1Fc was also effective against BT474 cells. To rule out potential artifacts caused by antibody aggregation, we further purified the monomer of SMET2.1Fc by using size-exclusion chromatography (Figure 2C, right panel) and then tested it with a small panel of human cancer cell lines expressing various levels of HER2 (breast cancer cell lines MCF7, SKBR3, and BT474, gastric cancer cell line NCI-N87, and ovarian cancer cell line SKOV3). Purified SMET2.1Fc monomer still contained a trace of aggregates (dimer) due to the relatively inefficient separation of the monomer from the aggregate during the purification process. In a stability study, incubation of the purified SMET2.1Fc monomer at 4 °C overnight or a long period (about 13 months) of storage at −80 °C did not lead to increased aggregation and degradation, suggesting generally high stability of the antibody (Figure 2C, right panel). Flow cytometry analysis showed that at a concentration of 10 nM, SMET2.1Fc bound to the cell lines as well as pertuzumab and slightly better than trastuzumab (Figure S2). The three antibodies stained all the cells strongly except MCF7, which is a HER2 low-expressing breast cancer cell line. Purified SMET2.1Fc monomer potently inhibited the growth of SKBR3 and BT474 with a potency much higher than that of trastuzumab and pertuzumab (Figure 4). SMET2.1Fc achieved a maximum inhibition of 60–70% while treatment with trastuzumab and pertuzumab only caused a maximum inhibition of approximately 20% at the concentrations tested. SMET2.1Fc was also more potent against SKBR3 than trastuzumab and pertuzumab in combination. Interestingly, SMET2.1Fc did not inhibit the growth of NCI-N87 and SKOV3 albeit with high HER2 expression; in contrast, weak inhibition was observed with trastuzumab and pertuzumab at high concentrations. None of the antibodies affected the growth of the human breast cancer cell line MCF7. In addition, neither a control VH-Fc nor SMET2.1 dAb had an effect on SKBR3 cells (Figure 4). 

### 3.4. Epitope Mapping of the Anti-HER2 dAbs

**Figure 5:**
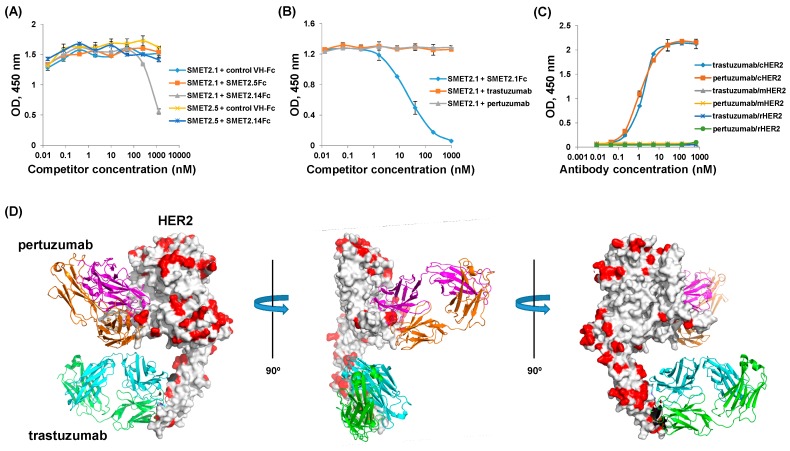
Epitope mapping of SMET2.1. (**A**) Competitive ELISA binding of the anti-HER2 dAbs. The concentrations of dAbs were kept constant (1.5 µg mL^−1^). The concentrations of competitors (Fc-fusion proteins) were fivefold serially diluted. Bound dAbs were detected by HRP-conjugated anti-FLAG tag antibody. (**B**) Competitive ELISA binding of SMET2.1 with IgG1 of trastuzumab and pertuzumab. The concentration of SMET2.1 was kept constant (2 µg mL^−1^). The concentrations of competitors (IgG1s or Fc-fusion proteins) were fivefold serially diluted. Bound SMET2.1 was detected by HRP-conjugated anti-FLAG tag antibody. (**C**) ELISA binding of trastuzumab and pertuzumab IgG1s to recombinant HER2 proteins from different species. Recombinant HER2 proteins were coated on 96-well plates at a concentration of 2 µg mL^−1^. Bound IgG1s were detected by HRP-conjugated goat anti-human IgG (Fc-specific) antibody. All the ELISA assays were performed in duplicate. Results are the mean ± standard deviation. (**D**) Superimposition of the crystal structures of hHER2-trastuzumab and hHER2-pertuzumab complexes. Variable amino acid residues of HER2 among mice, rats, cynomolgus monkeys, and humans are indicated in red.

In an attempt to elucidate the mechanisms underlying the substantially different antiproliferative activity and specificity between SMET2.1Fc and trastuzumab and pertuzumab, we combined biological and computational approaches to approximately localize their epitopes on HER2 (Figure 5). In an ELISA, we found that SMET2.1 competed with SMET2.14Fc while the two antibodies did not compete with SMET2.5 for binding to hHER2, suggesting that SMET2.1 and SMET2.14 bound to a similar area on HER2 and their epitopes did not overlap with that of SMET2.5 (Figure 5A). In another ELISA, SMET2.1 competed with SMET2.1Fc but not with trastuzumab and pertuzumab IgG1 for binding to hHER2, suggesting that the epitope of SMET2.1 did not overlap with those of trastuzumab and pertuzumab (Figure 5B). In yet another ELISA, we found that, in contrast to SMET2.1, both trastuzumab and pertuzumab bound only to hHER2 and cHER2 but not to mHER2 and rHER2 (Figure 5C). The existing crystal structures of trastuzumab-hHER2 and pertuzumab-hHER2 complexes allowed us to superimpose both trastuzumab and pertuzumab structures on a single hHER2 molecule (Figure 5D). By aligning the amino acid sequences of HER2 from all four species, we found that, indeed, trastuzumab and pertuzumab target HER2 surface areas that are variable among the species. Several highly conserved areas on hHER2 were identified that could provide sufficient contact for SMET2.1 without causing steric hindrance to trastuzumab and pertuzumab binding. Crystallization of the SMET2.1-hHER2 complex is underway, which could define precisely the epitope of SMET2.1. 

### 3.5. In Vivo Pharmacokinetics of SMET2.1Fc

**Figure 6:**
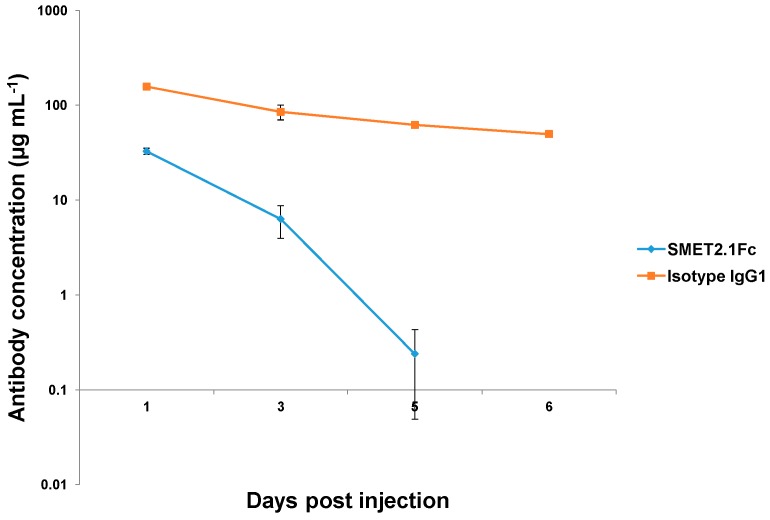
Pharmacokinetics of purified SMET2.1Fc monomer and an isotype IgG1 in NOD/SCID mice. Animals were intravenously injected with 0.5 mg antibody on day 0. Plasma was collected on days 1, 3, 5, and 6. Serum concentrations of antibodies were measured by ELISA. Each group included two animals. Plotted data are the mean ± standard deviation.

The pharmacokinetics of purified SMET2.1Fc monomer and an isotype human IgG1 antibody were tested in NOD/SCID mice. The control IgG1 antibody, which was specific for human EGFR, had high serum concentrations at all time points investigated (Figure 6). In contrast, SMET2.1Fc was rapidly cleared and serum concentrations dropped to an undetectable level after 6 days of infusion. 

## 4. Discussion

To date, three mAb-based therapeutics have been approved by the FDA to treat HER2-overexpressing cancers [9]. These include two mAbs, trastuzumab (Herceptin) and pertuzumab (Perjeta), which target the extracellular domains IV and II of HER2, respectively, and trastuzumab emtansine (Kadcyla), an antibody-drug conjugate (ADC) consisting of trastuzumab covalently linked to the cytotoxic agent emtansine. Although the introduction of HER2-directed therapies has improved the outcome of patients with breast and gastric cancers, the results of clinical trials in patients with other HER2-overexpressing cancers have been disappointing. Moreover, not all patients bearing the same type of cancer with high HER2 expression respond. A potential explanation is that some cancers become more dependent upon signaling from an individual pathway, such as HER2 for growth and proliferation, than other cancers [22]. On the other hand, however, the treatments cause toxicities in a significant percentage of patients—with cardiac dysfunctions and heart failures as the most severe ones—likely due to the expression of HER2 in normal tissues [23]. 

HER2 is a large transmembrane glycoprotein involved in multiple signal transduction pathways. It interacts preferentially with other ErbB members generating more potent signals than HER2 homodimerization. In addition, HER2 can partner with other membrane receptors such as insulin-like growth factor receptor 1 (IGF-1R) [24] and mucin 1 (MUC1) [25]. It is, therefore, conceivable that targeting different surface areas of HER2 might have varying impacts on the signaling networks leading to substantially different intervention efficacy and safety profiles. In this study, we identified a fully human VH-based dAb, SMET2.1, which, as an Fc-fusion protein, specifically inhibited the proliferation of HER2-overexpressing human breast cancer cells but not gastric and ovarian cancer cells. SMET2.1Fc had a potency against SKBR3 and BT474 cells higher than that of trastuzumab and pertuzumab (Figure 4). Interestingly, SMET2.1 bound to an epitope conserved in all four species and non-overlapping with those of trastuzumab and pertuzumab (Figure 3 and Figure 5). These results provide supporting evidence for the existence of unidentified surface areas on HER2 extracellular domains that are functionally more important for breast cancer tumorigenesis than those already known and can be targeted by antibody-based therapy. 

**Figure 7:**
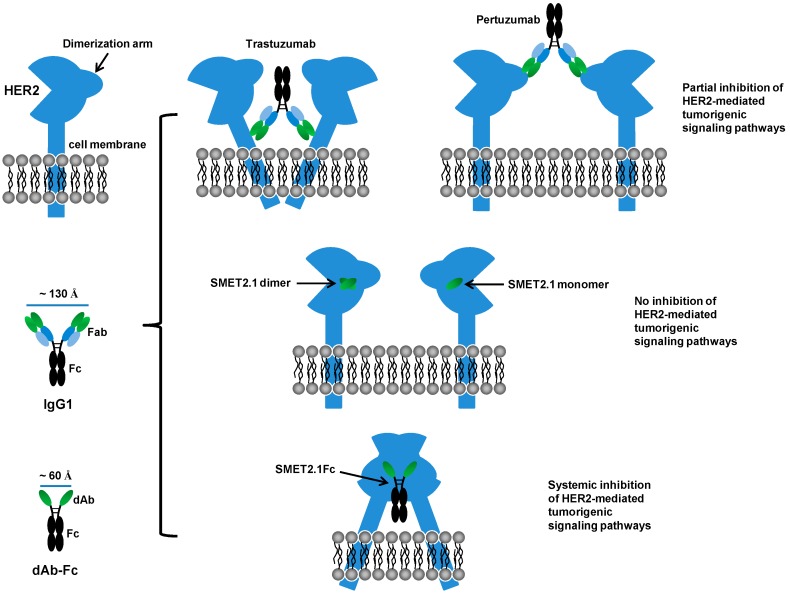
A potential molecular mechanism for potent inhibitory activity of SMET2.1Fc against HER2-overexpressing human breast cancer cells. The distance between antigen-binding sites of an IgG1 or a dAb-Fc fusion protein is estimated by using a published IgG1 crystal structure (Protein Data Bank entry 1HZH) as a template and the Measurement tool of the PyMOL molecular graphics system (version 1.5.0.4; Schrödinger, LLC).

The exact mechanisms by which SMET2.1 exerts its functions remain to be fully elucidated. Pertuzumab is directed against the extracellular domain II of HER2, which is required for the heterodimerization of HER2 with other ErbB members [11]. The antigen binding fragment (Fab) of pertuzumab directly inhibits HER2 association with its partner receptors, leading to the blockade of HER2-mediated signaling in addition to the ADCC activity mediated by Fc [12,26]. In contrast, the Fab of trastuzumab was ineffective in blocking the association of HER2 with HER3 in human breast cancer cells expressing low or high levels of HER2 [26] and, therefore, ADCC is the major mechanism of action of trastuzumab. We found that as a dAb, SMET2.1 did not inhibit the proliferation of SKBR3 cells, suggesting that cross-linking of HER2 is required for the antitumor activity of SMET2.1Fc (Figure 4). We speculate as a possible explanation for the remarkable inhibitory activity of SMET2.1Fc against HER2-overexpressing human breast cancer cells that because of the unique conserved epitope of SMET2.1 and the shorter distance between two antigen-binding sites of dAb-Fc compared to IgG1, cell-surface HER2 molecules could be locked into a rigid homodimer structure that results in systemic inhibition of HER2-mediated tumorigenic signaling pathways (Figure 7). Such an effect may not be so pronounced in other HER2-overexpressing cancer cells as in breast cancer cells because the former are not or less addicted to HER2 signaling. It is of note, however, that the purified SMET2.1Fc monomer still contains a trace of aggregates (dimer) (Figure 2C). Although the monomer preparation appeared to be stable and the ineffective control VH-Fc appeared to aggregate too (Figure S3), the potential influence of SMET2.1Fc dimer to the antitumor activity cannot be completely ruled out. SMET2.1 dAb also partially aggregated to form a dimer (Figure 1D) but did not inhibit the growth of SKBR3 cells (Figure 4). This result suggests that the dimer of SMET2.1 dAb may not be able to cross-link HER2 molecules on the cell surface due to a loss of HER2-binding activity or steric hindrance for binding to a second HER2 molecule, or the HER2-dAb complexation is ineffective against HER2 signaling (Figure 7). 

Some animals such as camels and sharks naturally produce unique antibodies devoid of light chains, designated heavy-chain antibodies (HCAbs). Compared to human VH sequences, the variable domains (V_H_H) of camelid HCAbs have amino acid substitutions in the FR regions, which might render V_H_H with additional ability to resist aggregation in the absence of light chain variable domains (VL) [27]. Certain human VH and VL family members are gifted with remarkable structural stability and can serve as FR scaffolds for displaying CDR diversity leading to large, valuable fully human dAb libraries for selection of antigen-specific high-affinity binders [20,21]. A previous study demonstrated the successful selection of two anti-HER2 dAbs from a phage-display human VH library, Gr3 and Gr6 [28]. An interesting finding is that albeit with the same FR sequence, Gr3 is monomeric whereas Gr6 exists as a tightly packed dimer, suggesting that CDR sequences play essential roles in Gr6 dimer formation. Crystallization of Gr6 revealed that, indeed, four amino acid residues on CDR3 loop, three of which are hydrophobic, and contribute to the dimerization. In agreement with this study, we found that SMET2.1, SMET2.5, and SMET2.14 with the same human VH3-23 germline-based FR sequence exhibit different oligomerization states (Figure 1D). Amino acid sequence alignment showed that the two groups of anti-HER2 dAbs have a high degree (87%) of sequence identity in the FR scaffold, but their CDR3 sequences vary in both length and composition (Table S1). Substitution of SMET2.1 CDR3 amino acid residues might help identify the determinants of the antibody dimerization. Exposure of the hydrophobic interface for VL or VH might also cause non-specificity of dAbs. SMET2.1 did not bind to several recombinant receptor proteins including sCD4, hEGFR, hHER3, and hHER4 in ELISA and CHO cells in flow cytometry (Figure 1 and Figure 3), but interaction with untested antigens remains possible. 

Favorable pharmacokinetics is a prerequisite for successful clinical development of antibody therapeutics. Antibody pharmacokinetics is partially determined by properties that include but are not limited to molecular size, stability, specificity, susceptibility to proteolysis, and neonatal Fc receptor (FcRn) binding activity [29,30]. SMET2.1Fc had a much shorter half-life than a control human IgG1 antibody in mice, likely due to a smaller molecular size and cross-reactivity with mouse HER2 or other mouse proteins that could act as a sink for rapid antibody clearance (Figure 6). Poor pharmacokinetics of SMET2.1Fc limits its potential for therapeutic use but the antibody could still be useful as an agent for the exploration of functionally important conserved structures on HER2 with implications for the design of novel therapeutics and elucidation of mechanisms of HER2-mediated tumorigenesis. 

## Supplementary Materials

The following are available online at https://www.mdpi.com/2073-4468/8/1/25/s1, Figure S1: Growth inhibition of HER2-overexpressing human breast cancer cell lines by the dAb-Fc fusion proteins in cell cultures; Figure S2: Comparative binding of SMET2.1Fc, trastuzumab and pertuzumab to HER2-expressing human cancer cell lines at a concentration of 10 nM as measured by flow cytometry; Figure S3: Preliminary characterization of a control VH-Fc fusion protein.
