# Supplementary material for: Human Domain Antibodies to Conserved Epitopes on HER2 Potently Inhibit Growth of HER2-Overexpressing Human Breast Cancer Cells In Vitro
Source: Antibodies (Basel). 2019 Mar 18;8(1):25. doi: 10.3390/antib8010025 (PMC6640707; doi:10.3390/antib8010025)
Supplement: Supplementary file 1 [file antibodies-08-00025-s001.pdf]

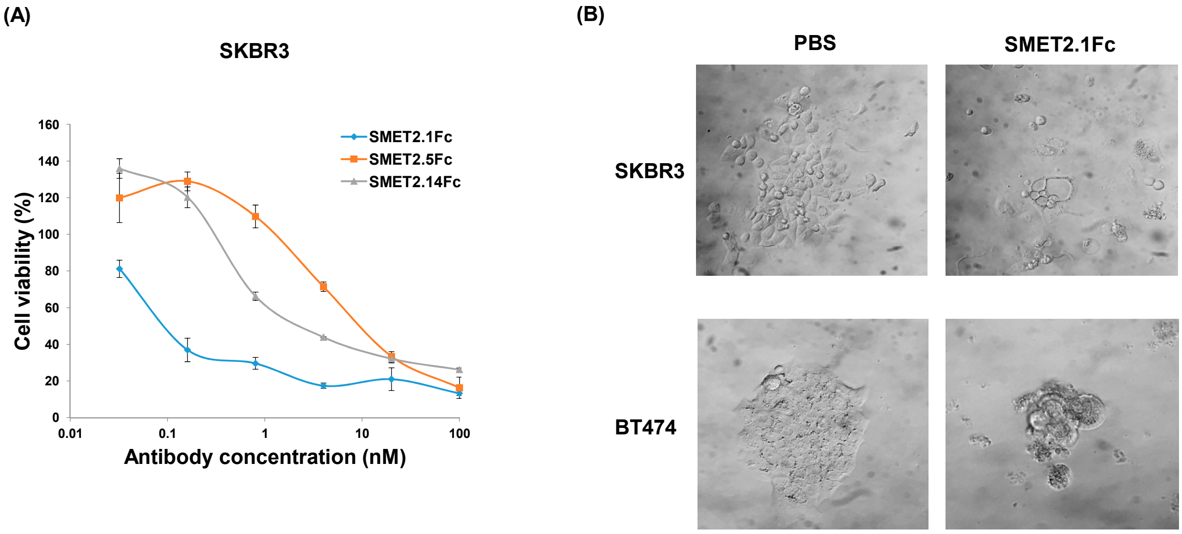

Figure S1. Growth inhibition of HER2-overexpressing human breast cancer cell lines by the dAb-Fc fusion proteins in cell cultures. (A) Dose-dependent inhibition of SKBR3 cells by the dAb-Fc fusion proteins. The assay was performed in duplicate. Results are means ± standard deviations. (B) Inhibition of SKBR3 and BT474 cells by SMET2.1Fc at a concentration of 4 nM. Representative images were taken after 5 days of incubation.

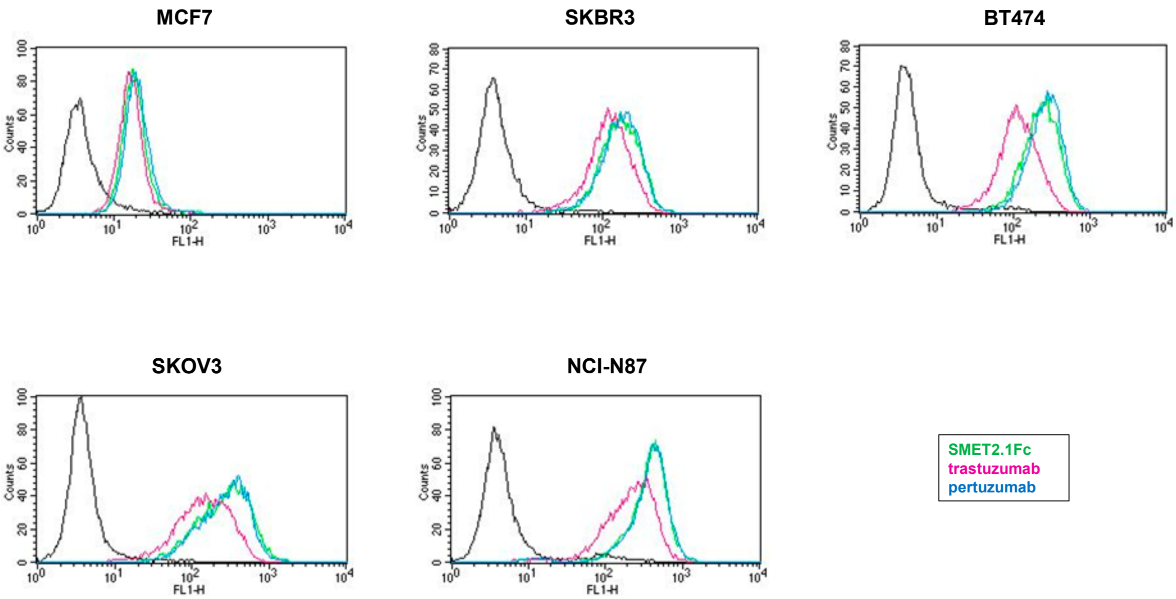

Figure S2. Comparative binding of SMET2.1Fc, trastuzumab and pertuzumab to HER2-expressing human cancer cell lines at a concentration of 10 nM as measured by flow cytometry. Trastuzumab and pertuzumab used in this experiment are full-length IgG1 antibodies. Diagrams for reference cells are in black. Diagrams for cells incubated with SMET2.1Fc, trastuzumab and pertuzumab are in green, pink and blue, respectively.

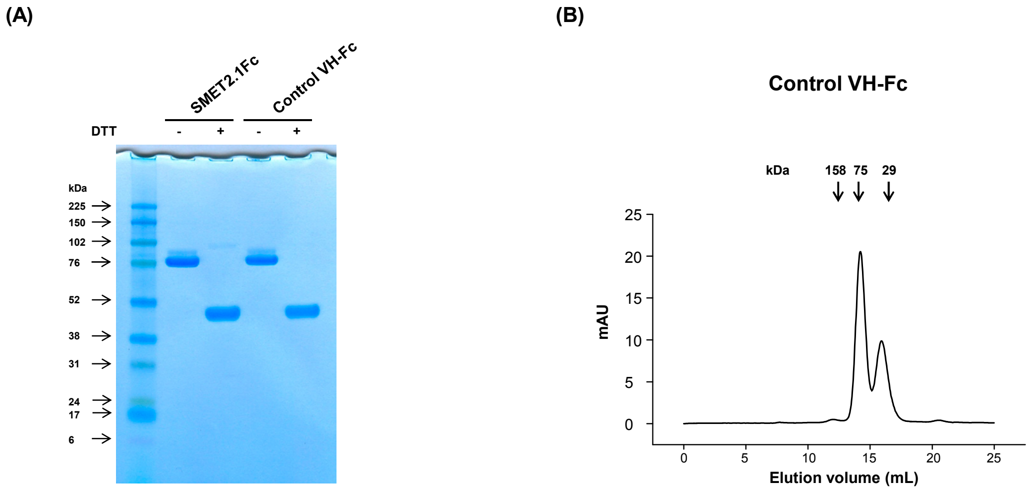

Figure S3. Preliminary characterization of a control VH-Fc fusion protein. (A) SDS-PAGE of the control VH-Fc fusion protein. Molecular masses of standards are shown on the left. (B) Size-exclusion chromatography of the control VH-Fc fusion protein. The arrows at the top indicate the elution volumes of molecular mass standards in PBS (pH7.4).

Table S1. Amino acid sequences of CDRs of anti-HER2 dAbs.

Antibody	CDR1[a]	CDR2	CDR3
SMET2.1	GFTFSSYA	IYTSGST	ATSPFFDY
SMET2.5	GFTFSDYY	IYYSGSI	ARADSSGYYYDIDY
SMET2.14	GFTVSSYS	IYTSGST	ARGWDLDY
Gr3	GFTVSYES	ISSSGGST	VTPERQCKQSTCYARPRY
Gr6	GVRLSAYD	ISSSGGST	VTLPDLCPGDNCTYPDAS

[a] CDR sequences are defined according to IMGT numbering scheme.
